# Causal effects of obstructive sleep apnea on chronic kidney disease and renal function: a bidirectional Mendelian randomization study

**DOI:** 10.3389/fneur.2024.1323928

**Published:** 2024-09-04

**Authors:** Yawei Hou, Yameng Li, Zhenwei Xiao, Zhenguo Wang

**Affiliations:** ^1^Institute of Chinese Medical Literature and Culture, Shandong University of Traditional Chinese Medicine, Jinan, China; ^2^The First Clinical Medical College, Shandong University of Traditional Chinese Medicine, Jinan, China; ^3^Department of Nephrology, Affiliated Hospital of Shandong University of Traditional Chinese Medicine, Jinan, China

**Keywords:** obstructive sleep apnea, chronic kidney disease, renal function, Mendelian randomization, causal relationship

## Abstract

**Background:**

Observational studies have suggested an association between obstructive sleep apnea (OSA), chronic kidney disease (CKD), and renal function, and vice versa. However, the results from these studies are inconsistent. It remains unclear whether there are causal relationships and in which direction they might exist.

**Methods:**

We used a two-sample Mendelian randomization (MR) method to investigate the bidirectional causal relation between OSA and 7 renal function phenotypes [creatinine-based estimated glomerular filtration rate (eGFRcrea), cystatin C-based estimated glomerular filtration rate (eGFRcys), blood urea nitrogen (BUN), rapid progress to CKD, rapid decline of eGFR, urinary albumin to creatinine ratio (UACR) and CKD]. The genome-wide association study (GWAS) summary statistics of OSA were retrieved from FinnGen Consortium. The CKDGen consortium and UK Biobank provided GWAS summary data for renal function phenotypes. Participants in the GWAS were predominantly of European ancestry. Five MR methods, including inverse variance weighted (IVW), MR-Egger, simple mode, weighted median, and weighted mode were used to investigate the causal relationship. The IVW result was considered the primary outcome. Then, Cochran’s Q test and MR-Egger were used to detect heterogeneity and pleiotropy. The leave-one-out analysis was used for testing the stability of MR results. RadialMR was used to identify outliers. Bonferroni correction was applied to test the strength of the causal relationships (*p* < 3.571 × 10^−3^).

**Results:**

We failed to find any significant causal effect of OSA on renal function phenotypes. Conversely, when we examined the effects of renal function phenotypes on OSA, after removing outliers, we found a significant association between BUN and OSA using IVW method (OR: 2.079, 95% CI: 1.516–2.853; *p* = 5.72 × 10^−6^).

**Conclusion:**

This MR study found no causal effect of OSA on renal function in Europeans. However, genetically predicted increased BUN is associated with OSA development. These findings indicate that the relationship between OSA and renal function remains elusive and requires further investigation.

## 1 Introduction

Chronic kidney disease (CKD), distinguished by structural and functional impairments of the kidneys, is generally diagnosed when the Estimated Glomerular Filtration Rate (eGFR) drops below 60 mL/min per 1.73 m^2^ or in instances where kidney damage persists for a minimum of 3 months (1). Being a significant global health issue, CKD affects an estimated 700 million individuals globally (2). It is projected that by 2040, CKD will ascend to become the fifth primary cause of death worldwide (2). There is currently no cure for CKD, and the primary emphasis in treatment and management revolves around modifying risk factors and controlling complications. As CKD progresses, it invariably results in a consistent decline in renal function, frequently necessitating renal replacement therapy for patients suffering from End-Stage Kidney Disease (ESKD) (3, 4). This situation imposes a substantial economic burden on both societal and familial fronts owing to medical expenditures (5). Therefore, there is an exigent requirement to discern treatable risk factors connected with the onset and advancement of CKD.

Obstructive Sleep Apnea (OSA), a condition with high prevalence, is experienced by an estimated 38% of the global adult population in its moderate-to-severe forms (6). OSA is marked by the repeated closure of the upper airway during sleep, consequentially causing sleep fragmentation and intermittent hypoxia (7). Numerous observational studies offer evidence supporting a bidirectional relationship between OSA and CKD (8, 9). OSA may heighten the risk of renal damage, CKD can reciprocally impose a heightened risk of OSA (10–17). Nevertheless, the results derived from observational studies have been inconsistent. For example, in individuals afflicted with Coronary Artery Disease, the severity of OSA was not independently associated with CKD (18). Another study illustrated that OSA alone does not constitute a risk factor for CKD. However, for patients presenting with Metabolic Syndrome, OSA served as an additional burden escalating the risk of CKD (19).

Traditional observational studies are limited in their ability to completely eliminate confounding bias or reverse causality (20). The assessment of causality between OSA and CKD based on the associations observed in observational studies is challenging. Randomized controlled trials are less susceptible to confounding; however, conducting such trials to evaluate the effects of potentially harmful exposures like OSA would be unethical or impractical. Elucidating the causality between OSA and CKD is crucial as it provides insights into the underlying biological mechanisms of the disease and aids in the development of therapeutic strategies for improving CKD prevention. The Mendelian Randomization (MR) design serves as a valuable technique in epidemiological studies for assessing causal inference by employing genetic variants as instrumental variables (21). The strength of MR lies in the random assignment of genetic variants from parents to offspring, which are impervious to self-selective behavior—this can fortify the causal inference by mitigating potential unmeasured residual confounding and precluding reverse causality (22). MR analysis capitalizes on genetic variations as Instrumental Variables (IVs) to corroborate causal associations, taking advantage of their diminished susceptibility to measurement errors or biases. Two-sample MR (TSMR) is commonly applied to link exposure and outcome data sourced from distinct Genome-Wide Association Study (GWAS) datasets (23). For this project, bidirectional MR analyses were employed to surmount the limitations intrinsic to observational studies and to probe into the relationship between OSA, and CKD, renal function.

## 2 Methods

### 2.1 Study design

A TSMR approach was utilized employing summary statistics from distinct GWAS for OSA and CKD. Initially, a forward MR analysis was undertaken to explore the associations between genetically predisposed OSA and both CKD and renal function. Subsequently, given the potential influence of impaired renal function on OSA, a reverse MR analysis was executed to scrutinize the associations between genetically influenced renal function and OSA. A robust MR framework adheres to three critical assumptions: (1) instrumental variables (IVs) are strongly associated with the exposure; (2) IVs are not related to any confounders influencing both exposure and outcome; and (3) the influence of IVs on outcomes is only via their effect on exposure rather than any other causal pathways (24). This article solely employed summary data. The original studies have obtained the necessary ethical approval and informed consent from patients.

### 2.2 Genetic associations with OSA

The full GWAS summary statistics pertaining to OSA were extracted from the most recent published data in the FinnGen database, which included 375,657 participants—38,998 patients and 336,659 controls (25). The diagnosis of OSA was made based on the International Classification of Diseases, Tenth Revision (ICD-10) and Ninth Revision (ICD-9) codes (ICD-10: G47.3, ICD-9: 3472). These were acquired from the Finnish National Hospital Discharge Registry and the Causes of Death Registry. This diagnosis was established on the basis of subjective symptoms, clinical examination, and sleep registration applying an apnea-hypopnea index ≥5 events·h^−1^ or a respiratory event index ≥5 events·h^−1^. By amalgamating ICD codes from various registries, we constituted disease endpoints. In the fifth round of data from FinnGen, the prevalence of OSA was 7.69%, with 63% of OSA patients being male. The average age of the OSA group was 58.9 ± 13.3 years, with a BMI of 31.72 ± 6.74 kg/m^2^. The age at OSA diagnosis was 55.3 ± 11.9 years. In contrast, the average age in the non-OSA group was 51.8 ± 17.7 years, with a BMI of 26.87 ± 5.02 kg/m^2^, while the overall average BMI was 27.25 ± 5.34 kg/m^2^. Compared with the non-OSA group, the OSA group had a higher prevalence of diseases such as type 2 diabetes, hypertension, and coronary heart disease. Age, sex, and the 10 first principal components were adjusted as covariates in the original GWAS study (26).

### 2.3 Genetic associations with CKD and renal function

There are seven phenotypes included, and they are primarily from Chronic Kidney Disease Genetics (CKDGen) Consortium and UK Biobank: creatinine-based estimated glomerular filtration rate (eGFRcrea), cystatinC-based estimated glomerular filtration rate (eGFRcys), blood urea nitrogen (BUN), urine albumin to creatinine ratio (UACR), CKD (defined as an estimated glomerular filtration rate (eGFR) of less than 60 mL/min/1.73 m^2^), rapid decline of eGFR (Rapid3) (the eGFR decreases by more than 3 mL/min/1.73 m^2^ per year), and rapid progress to CKD (CKDi25) (defined as the decrease of eGFR ≥25% of baseline accompanied by the progression from no CKD to CKD). Instrument variable summary statistics for CKD were sourced from a meta-analysis conducted by the CKDGen Consortium, which incorporated 23 European ancestry cohorts (*n* = 480,698; 41,395 patients and 439,303 controls) (27). Individuals of European ancestry in the CKDGen dataset had a mean age of 54 years old, and 50% of them were male, with a median eGFR of 91.4 mL/min/1.73 m^2^ and a prevalence of CKD of 9%. All genetic associations were adjusted for sex, age, study site, genetic principal, components, relatedness, and other study-specific features. The GWAS summary statistics for eGFRcrea, eGFRcys, and BUN were sourced from a meta-analysis that included data from the CKDGen Consortium and the UK Biobank, encompassing 1,201,909 participants (28). The UACR data were derived from a separate meta-analysis, which documented the summary data from both trans-ethnic (*n* = 564,257) and European-ancestry populations (*n* = 547,361) (29). Summary statistics for Rapid3 (comprising 34,874 cases and 107,090 controls) and CKDi25 (encompassing 19,901 cases and 175,244 controls) were obtained from a meta-analysis of 42 GWAS studies from the CKDGen Consortium and the UK Biobank (30). For detailed diagnostic criteria and inclusion procedures, please refer to the original literature. The datasets for CKD, eGFR, UACR, Rapid3, and CKDi25 are accessible at http://ckdgen.imbi.uni-freiburg.de/. Detailed information about each dataset can be found in Supplementary Table S1.

### 2.4 Selection of instrumental variables (IVs)

First, we procured Single Nucleotide Polymorphisms (SNPs) that were strongly associated (*p* value <5 × 10^−8^) with exposures in each MR analysis. For CKDi25 and Rapid3, where only a few significant SNPs were found using the *p* < 5 × 10^−8^ threshold, SNPs were selected as IVs at *p* < 5× 10^−6^. Second, it is crucial to ensure the chosen IVs satisfy the independence criterion. To evaluate the independence of these variables and account for potential linkage disequilibrium effects, a linkage disequilibrium parameter (R^2) threshold of 0.001 and a genetic distance cutoff of 10,000 kb were implemented. Additionally, during the reverse MR analysis, duplicate values from the seven renal function phenotype IVs were eliminated. Third, Phenoscanner (31) was employed to check potential confounding factors (such as hypertension, obesity, overweight, diabetes, among others) that might be related to the IVs, thus preventing such factors from interfering with the impact of exposure on outcomes. Moreover, IVs associated with the outcomes at a significance level of *p* < 5 × 10^−8^ were excluded. We harmonized the effect alleles of outcome-associated SNPs to ensure consistency with those of exposure-associated SNPs, taking into account allele letters and frequencies. Also, palindromic SNPs were excluded from the analysis. To further bolster the reliability of our research results, we applied Steiger filtering to remove SNPs that exhibited a stronger correlation with the outcomes than with the exposures (32). The meticulous selection process for IVs as described above significantly enhances the credibility of our findings. Furthermore, to eliminate bias induced by weak IVs in the results, we computed the F statistic. The F statistic is calculated using the formula F = R^2^ (n-k-1)/[k (1-R^2^)], where R^2^ signifies the extent to which the IVs explain the exposure.

### 2.5 Mendelian randomization analysis

To investigate the causal relationship between exposure and outcome, several methods were utilized, including Inverse Variance Weighted (IVW), MR-Egger, Weighted Median, Simple Mode, and Weighted Mode. The point estimates obtained through IVW correspond to a weighted linear regression of SNP-outcome associations against SNP-exposure associations, with no regard to intercept. It is imperative when using the IVW method to ensure the absence of pleiotropy among SNPs, as this can significantly bias the results (33). In contrast, the MR-Egger method assumes the Instrument Strength Independent of Direct Effect (InSIDE) assumption and primarily examines the dose–response relationship between IVs and outcomes (34). This method takes into account the presence of pleiotropy to a certain extent. Even if most IVs have pleiotropy, MR Egger can provide effective estimates (34).

The weighted median method is effective in mitigating the impact of using invalid IVs and can provide consistent estimates of causal effects, even when up to 50% of the information is derived from genetic variations in invalid instruments (35). In contrast, weighted mode methods exhibit lower capability in detecting causal effects but are associated with fewer biases (36). If there is no pleiotropy, we chose IVW as the primary method for conducting our MR analysis. If pleiotropy exists, MR-Egger will be employed as the main method, along with the direction of effect size in four MR methods.

### 2.6 Sensitivity analysis

In our study, we utilized Cochran’s Q test to evaluate heterogeneity. Specifically, the inter-instrument Q-test was applied to probe heterogeneity arising from multiplicity or other factors (37). To identify pleiotropy, we conducted the MR-Egger regression test. A significant deviation of the intercept term from zero indicates the presence of horizontal pleiotropy (34). In instances where heterogeneity or horizontal pleiotropy was detected, estimates were recalculated using IVW, MR-Egger, and other methods after the removal of outlier SNPs identified through Radial MR analysis (38). Additionally, a leave-one-out analysis was performed, whereby SNPs were systematically removed one at a time, and the effect was recomputed.

### 2.7 Statistical analysis

All statistical analyses were conducted using R (version 4.2.3, R Foundation for Statistical Computing, Vienna, Austria). MR analyses were carried out using the “TwoSampleMR” and “RadialMR” packages. To control the type I error rate in the multiple testing, we utilized the Bonferroni correction method. This procedure involves dividing the critical significance level by the number of tests conducted, providing a simple yet effective way to manage multiple comparisons. However, it’s worth noting that such correction methods can become overly conservative when a large number of tests are performed (39). In our study, Bonferroni correction was applied to account for multiple testing of the associations between OSA and 7 renal function outcomes (i.e., eGFRcrea, eGFRcys, BUN, UACR, Rapid3, CKDi25, and CKD) with 5 methods. A two-sided *p*-value of <3.571 × 10^−3^[0.05/(1 × 2 × 7)] was considered significant.

## 3 Results

### 3.1 Forward MR analysis

**Figure 1:**
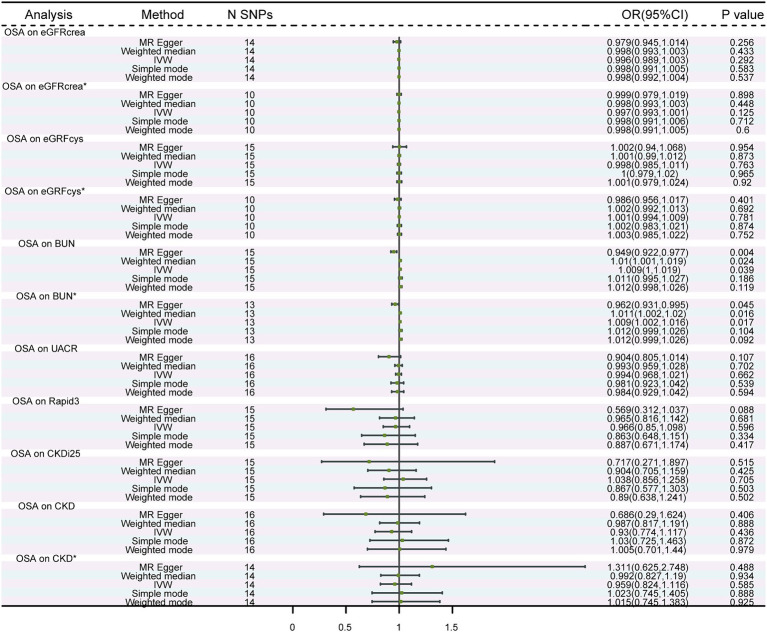
MR analysis of the causality of OSA on renal function. OSA, obstructive sleep apnea; eGFRcrea, creatinine-based estimated glomerular filtration rate; eGFRcys, cystatinC-based estimated glomerular filtration rate; BUN, blood urea nitrogen; UACR, urine albumin to creatinine ratio; Rapid3, rapid decline of eGFR; CKDi25, rapid progress to CKD; CKD, chronic kidney diseases. IVW, Inverse Variance Weighted; ^*^The results of after removing the outlier SNPs.

The number of independent SNPs selected as IVs for OSA was 14 for the analysis of association with eGFRcrea, 15 with eGFRcys, 15 with BUN, 16 with UACR, 15 with Rapid3, 15 with CKDi25, and 16 for CKD. We employed a more stringent *p*-value criterion in our analysis. A two-sided *p* value of <3.571 × 10^−3^ was considered significant. The primary two-sample MR analysis showed no significant association between genetically determined OSA and renal function phenotypes. These results were reproduced in the other analysis methods (Figure 1). Heterogeneity was suspected regarding the association between OSA and eGFRcrea, eGFRcys, BUN, CKD. The MR-Egger intercept, which is an indicator of genetic pleiotropy, was statistically significant for OSA between BUN (Supplementary Table S2). The scatter plots were shown in Supplementary Figures S1–S2. The results of leave-one-out sensitivity and single SNP risk analysis were shown in Supplementary Figures S3–S6. The heterogeneity or horizontal pleiotropy was noted in OSA between eGFRcrea, eGFRcys, BUN, CKD, we recomputed IVW, MR-Egger and other methods estimates after removing the outlier SNPs identified by Radial MR. The MR analysis showed no significant association between genetically determined OSA and eGFRcrea, eGFRcys, BUN, CKD (Figure 1). There was no evidence of significant heterogeneity. There was still pleiotropy between OSA and BUN (Supplementary Table S3). At this point, we selected MR-Egger as the primary analysis method and, combined with the direction of effect sizes from other methods, we did not find a significant association between OSA and BUN.

### 3.2 Reverse MR analysis

**Figure 2:**
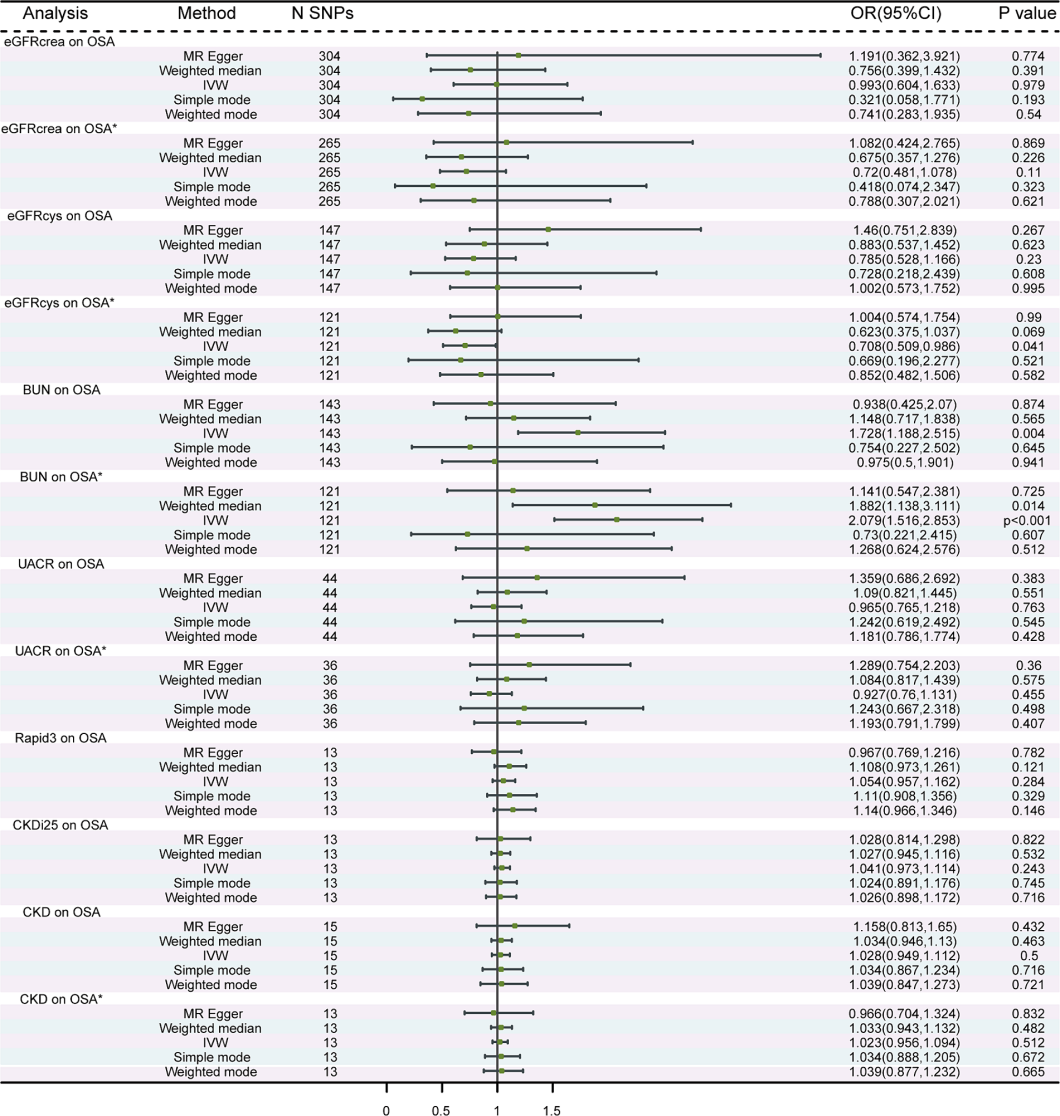
MR analysis of the causality of renal function on OSA. OSA, obstructive sleep apnea; eGFRcrea, creatinine-based estimated glomerular filtration rate; eGFRcys, cystatinC-based estimated glomerular filtration rate; BUN, blood urea nitrogen; UACR, urine albumin to creatinine ratio; Rapid3, rapid decline of eGFR; CKDi25, rapid progress to CKD; CKD, chronic kidney diseases. IVW, Inverse Variance Weighted; ^*^ The results of after removing the outlier SNPs.

A total of 304, 147, 143, 44, 13, 13, and 15 independent SNPs were selected as IVs for the MR analysis investigating the associations of eGFRcrea, eGFRcys, BUN, UACR, Rapid3, CKDi25, and CKD with OSA, respectively. The IVW method was employed, revealing suggestive evidence of a potential causal association between BUN and the risk of OSA (*p* = 0.004) (Figure 2). Heterogeneity was observed in the associations between eGFRcrea, eGFRcys, BUN, UACR, CKD, and OSA. Additionally, horizontal pleiotropy was detected in the relationships between eGFRcys and OSA (Supplementary Table S2). At this point, using the MR-Egger method as the primary analysis method, we did not find a significant association between eGFRcys and OSA. This result was also supported by four other methods. The scatter plots were shown in Supplementary Figures S7–S8. The results of leave-one-out sensitivity and single SNP risk analysis were shown in Supplementary Figures S9–S16. After excluding outlier SNPs, we recalculated the estimates using IVW, MR-Egger, and other methods. We discovered a significant association between BUN and OSA using the IVW method (OR: 2.079; 95% CI: 1.516–2.853; *p* = 5.72 × 10^−6^) (Figure 2). Importantly, there was no evidence of significant heterogeneity or pleiotropy in the associations (Supplementary Table S3).

## 4 Discussion

Our findings indicated that OSA did not directly lead to CKD, which contradicts prior observational studies. Furthermore, the lack of a genetic correlation between OSA and different renal function phenotypes supported this result. However, in our reverse MR analysis, we observed a positive correlation between BUN and OSA. Based on our findings, an elevation in BUN levels could be associated with an increased risk of OSA.

Contrary to our study results, recent epidemiological research has established a link between OSA and CKD. For instance, a cohort study involving more than 3 million US veterans—predominantly males with a mean age of 60.5 years—indicated that an onset diagnosis of OSA was associated with a higher incidence of CKD and a more rapid decline in kidney function over time as compared to those without OSA (40). In a retrospective and longitudinal population-based cohort study leveraging the Taiwan Longitudinal Health Insurance Database 2000, it was observed that individuals with OSA demonstrated an elevated risk of developing CKD, even after excluding those with hypertension and diabetes. The adjusted OR for this association was 1.37. A subgroup analysis further revealed a higher incidence of CKD among women with OSA. However, no significant correlation was found between OSA and CKD development in men (41). A study involving older participants aged 65 years and above, recruited from the general population, involved overnight polysomnography for 277 individuals. The results indicated an increased risk of rapid kidney function decline over an 11-year follow-up period among those with an Apnea-Hypopnoea Index (AHI) of 30 or higher. These findings remained statistically significant even when adjusted for various factors, such as age, sex, BMI, smoking status, diabetes mellitus, hypertension, and history of cardiovascular disease. Thus, it can be inferred that AHI serves as an independent risk factor for glomerular kidney function decline (42). Yayan et al. (43) concluded that CKD is more prevalent in patients with OSA compared to those without OSA, and the frequency of CKD escalates as the severity of OSA intensifies. Moreover, Marrone suggested that severe hypoxia, even if experienced for a limited duration during the night, may pose a greater risk factor for renal damage in patients with OSA than average SpO_2_ levels and/or frequency of apnea events (15). Chang et al. (16) reported that severe OSA independently increases the risk of CKD. Additionally, a study conducted by Adams et al. disclosed a correlation between OSA and stages 1–3 of CKD (17). A cross-sectional study further highlighted the prevalence of OSA in non-dialysis CKD patients to be 28%, with an incidence rate of 88%. The study also demonstrated a rise in the risk and severity of OSA as CKD stages progressed (14). The available real-world data indicates a reciprocal relationship between OSA and CKD. It suggests that individuals with OSA may have an increased likelihood of developing CKD and experiencing a decline in kidney function. Conversely, patients with CKD are more vulnerable to developing OSA. However, it is essential to acknowledge that these observational studies have inherent limitations, including methodological shortcomings, small sample sizes, selection bias, and inadequate adjustment for confounding factors, which prevent the establishment of a definitive causal link.

However, it’s worth noting that not all observational studies found a definitive significant link between OSA, CKD, and renal function. For instance, a retrospective cohort sub-study of the Wisconsin Sleep Cohort Study did not identify any association between the severity of sleep apnea and the decline in renal function (44). Lee et al. (19) suggested that OSA alone does not pose a risk factor for CKD, but in patients with metabolic syndrome, OSA was an additional burden escalating the risk of CKD. Also, Fernandes et al. (45) found a high prevalence—approximately 67%—of OSA in patients with stages 3b-4 CKD. However, intriguingly, the AHI was very similar between these two groups of patients, and no significant association was discerned between AHI and the eGFR. The retrospective study conducted by Uyar et al. (46) assessed patients diagnosed with OSA, excluding those with a previous diagnosis of CKD. The results showed no difference between OSA patients and the control group when evaluated concerning an estimated eGFR of less than 60 mL/min/1.73m^2^. Moreover, no correlation was observed between eGFR and the desaturation index. The study conducted by Canales et al. did not establish a significant relationship between renal function and sleep-disordered breathing (47). Furthermore, OSA was not found to be an identifying factor for patients at risk of CKD (48).

Due to the limitations of association studies in addressing causality, it remains challenging to definitively establish the causal relationship between OSA and CKD based solely on observational studies. Therefore, it is important to interpret the aforementioned findings with caution. Contrary to the majority of observational studies, our investigation did not uncover any causal link between OSA and CKD. In the reverse MR analysis, the results indicated that BUN has a causal relationship with OSA. However, there was no evidence of a causal relationship between CKD, as well as other renal function phenotypes, and OSA. There are a couple of possible reasons that may contribute to the association between OSA, CKD and renal function in observational studies. There are several potential factors that could contribute to the observed association between OSA and CKD in observational studies. Age, sex, diabetes, hypertension, glomerulonephritis, cholesterol and cigarette smoking are established risk factors for CKD (49). CKD patients with cardiovascular disease, diabetes, smoking habit and higher serum phosphorus have a higher risk of kidney damage (50). And in patients with non-dialysis CKD, the cardiovascular risk increases linearly with the higher levels of LDL cholesterol (51). OSA is also a recognized risk factor for cardiovascular disease. Patients with OSA often exhibit comorbidities such as hypertension, diabetes, obesity, and cardiovascular disease (52–54). OSA may be linked to CKD through shared conditions like obesity, hypertension, and diabetes, but the exact influence of each condition is difficult to determine (55–57). In the reverse MR analysis, we found an association between BUN and OSA. The increase in BUN usually occurs when the glomerular filtration rate decreases by more than 50%, which means that in CKD patients, an increase in BUN often represents the disease progressing to a later stage. Contemporary research elucidates that in patients undergoing hemodialysis, BUN exhibits a significant correlation with OSA (58–61). Moreover, multiple studies have demonstrated that optimized dialysis therapy can mitigate the severity of sleep apnea in patients afflicted with End-Stage Renal Disease (ESRD) (62–64). This implies that ESRD may elevate the risk of OSA. Present-day research has suggested various pathophysiological mechanisms through which ESRD could precipitate OSA, encompassing neuropathy or myopathy induced by uremia and hypervolemia. Diminished sensory function and denervation of the Upper Airway (UA) dilator muscle have been demonstrated to contribute to the pathogenesis of UA obstruction in patients diagnosed with OSA (65). In ESRD, uremic neuropathy is prevalent and may impinge on the sensory function of the UA, thereby augmenting UA collapsibility (66). Moreover, uremic myopathy, known to exacerbate the fatigability of the respiratory muscles (67), could potentially result in decreased tone of the UA dilator muscles, leading to an ensuing increase in UA collapsibility during sleep. On the other hand, there exists a substantial and well-established body of evidence underscoring the role of fluid overload in the pathogenesis of sleep apnea, particularly in conditions typified by fluid overload such as heart failure and End-Stage Renal Disease (ESRD) (68, 69). Hypervolemia and the rostral fluid shift from the legs overnight can both contribute to subsequent fluid accumulation in the neck. This accumulation can result in a reduction in the cross-sectional area of the UA and an increased collapsibility, thereby predisposing individuals to OSA (68). It is also plausible that fluid overload contributes to OSA not merely through its impact on UA collapsibility, but also potentially by influencing ventilatory instability (70). Other research has suggested a direct and independent correlation between the degree of fluid overload and the severity of OSA in ESRD (71–73).

Our MR study offers several key advantages. Firstly, to the best of our knowledge, it is the first study to assess the causal relationship between OSA and CKD, as well as renal function, using a two-sample MR analysis. Second, we utilized GWAS datasets predominantly from populations of European ancestry to mitigate the effects of population stratification. Third, different estimation models and rigorous sensitivity analysis were used to ensure the reliability and robustness of the results. However, our study has certain limitations. Firstly, the exclusive inclusion of participants with European ancestry in our dataset introduces potential participant overlap, and the generalizability of the results to the entire population needs further verification. Secondly, despite implementing a rigorous process to identify outlier variants and mitigate horizontal pleiotropy, complete elimination of its impact was unattainable due to the complex and uncertain biological functions of numerous genetic variants. Thirdly, larger sample sizes and more advanced methodologies are required to confirm the findings and comprehensively demonstrate statistical power. Finally, our study did not conduct subgroup analyses. In our analysis, the definitions of OSA and CKD were based on binary variables (i.e., the presence or absence of the disease) without considering the severity of these conditions. This could lead to an incomplete understanding of the relationship between OSA and CKD. For instance, if only severe OSA significantly increases the risk of CKD, while mild or moderate OSA has a lesser or no impact, our analysis may fail to capture this distinction. Additionally, the stages of CKD progression could also affect its association with OSA, but due to the lack of data, we were unable to assess this variation.

In conclusion, our MR analysis indicates that genetically predicted OSA does not have a causal impact on CKD and renal function phenotypes. This finding contradicts the results of most observational studies. Additionally, in the reverse MR analysis, only BUN was found to be statistically associated with OSA. To ensure the accuracy of our results, future research should rely on higher quality GWAS data and utilize more advanced methods. Furthermore, this study emphasizes the importance of further investigating the underlying mechanism linking OSA and CKD.

## Data Availability

The original contributions presented in the study are included in the article/Supplementary material, further inquiries can be directed to the corresponding author.
