# Supplementary material for: Causal effects of obstructive sleep apnea on chronic kidney disease and renal function: a bidirectional Mendelian randomization study
Source: Front Neurol. 2024 Sep 4;15:1323928. doi: 10.3389/fneur.2024.1323928 (PMC11408330; doi:10.3389/fneur.2024.1323928)
Supplement: Supplementary file 1 [file Table_1.DOCX]

**Supplementary Figures**


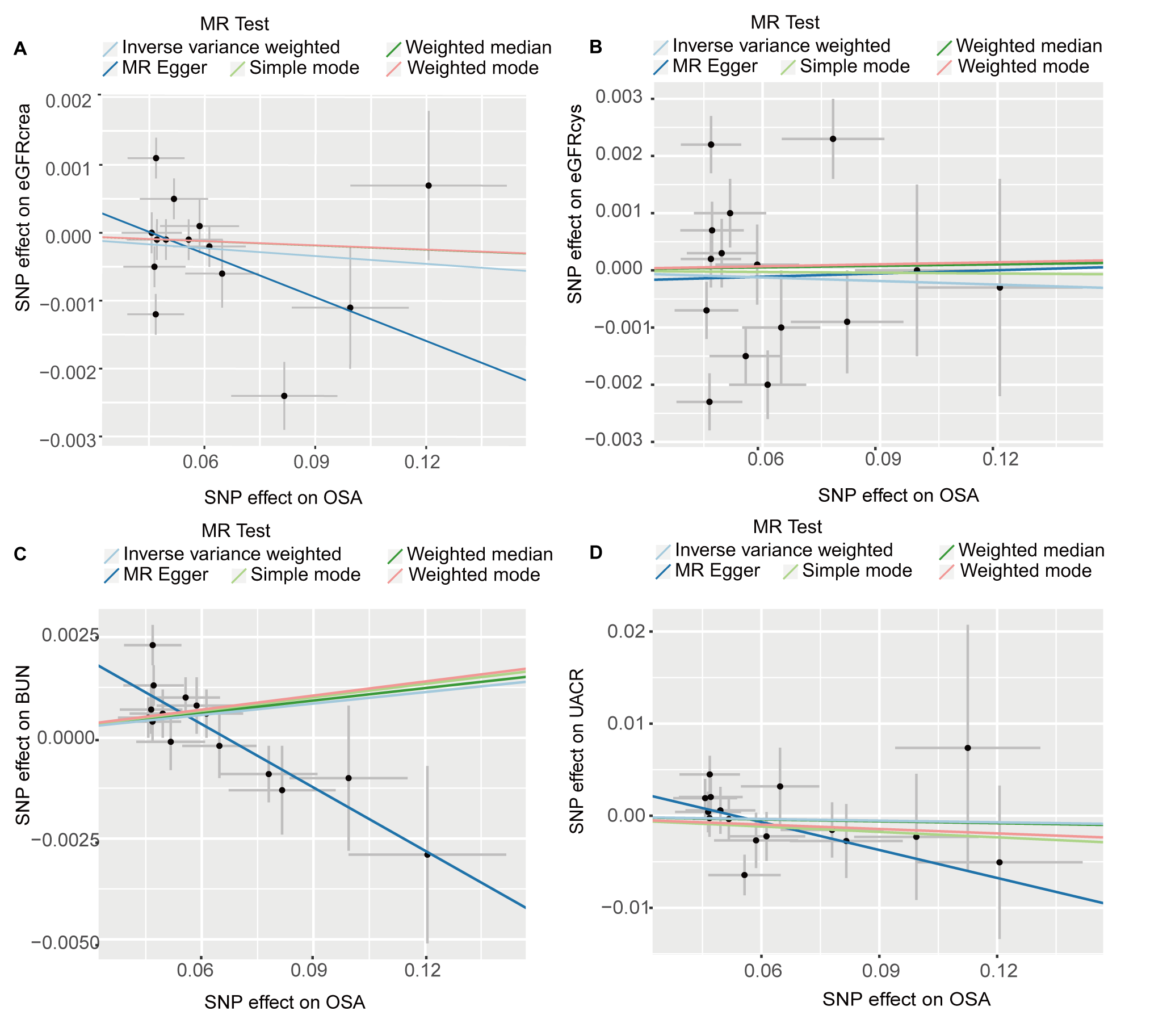


Supplement Figure 1. Scatter plot of the Mendelian randomization analysis results for the effect of the OSA on renal function phenotypes. (A) Causal effect of OSA on eGFRcrea using different MR methods; (B) Causal effect of OSA on eGFRcys using different MR methods; (C) Causal effect of OSA on BUN using different MR methods. (D) Causal effect of OSA on UACR using different MR methods.


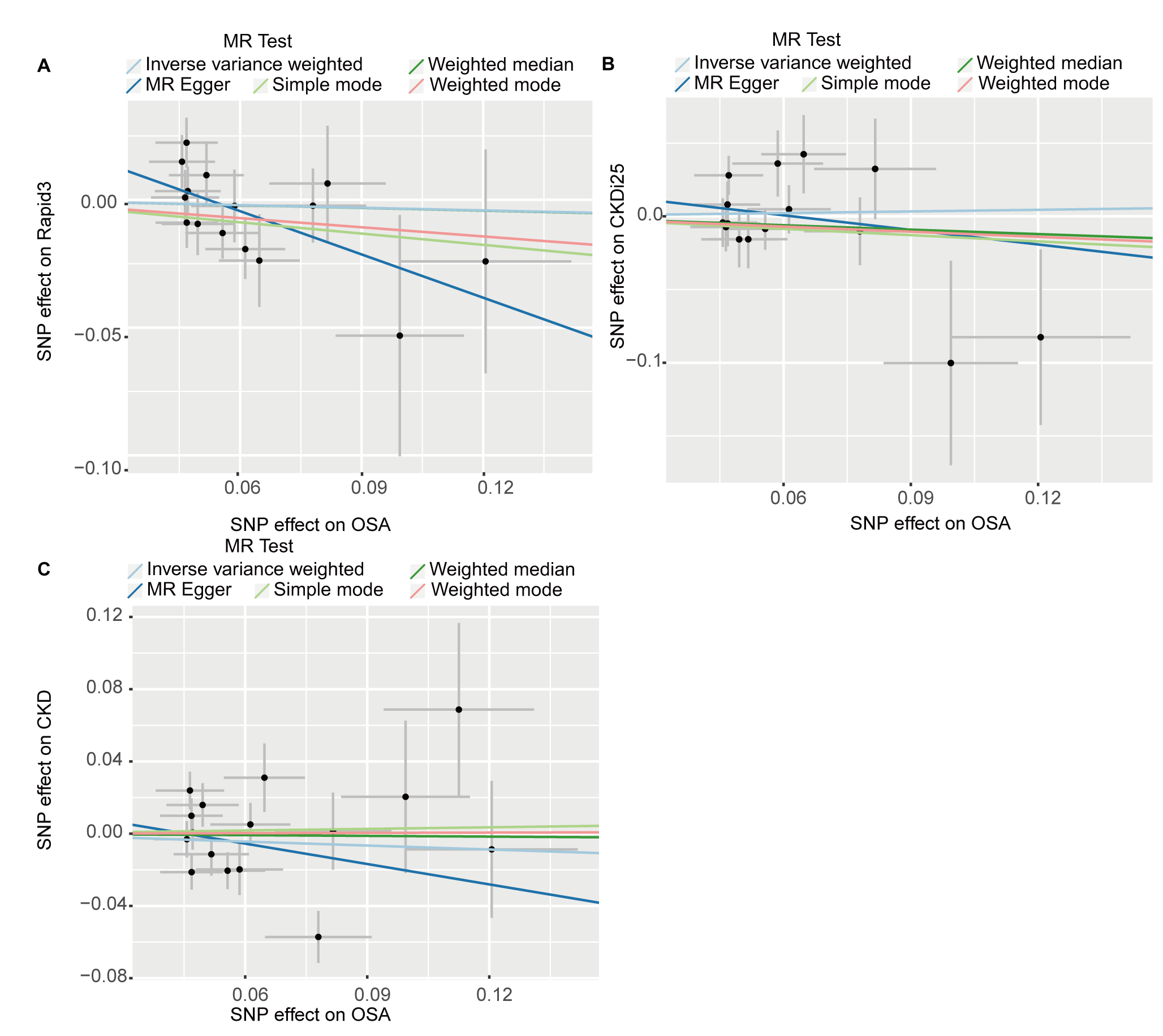


Supplement Figure 2. Scatter plot of the Mendelian randomization analysis results for the effect of the OSA on renal function phenotypes. (A) Causal effect of OSA on Rapid3 using different MR methods; (B) Causal effect of OSA on CKDi25 using different MR methods; (C) Causal effect of OSA on CKD using different MR methods.


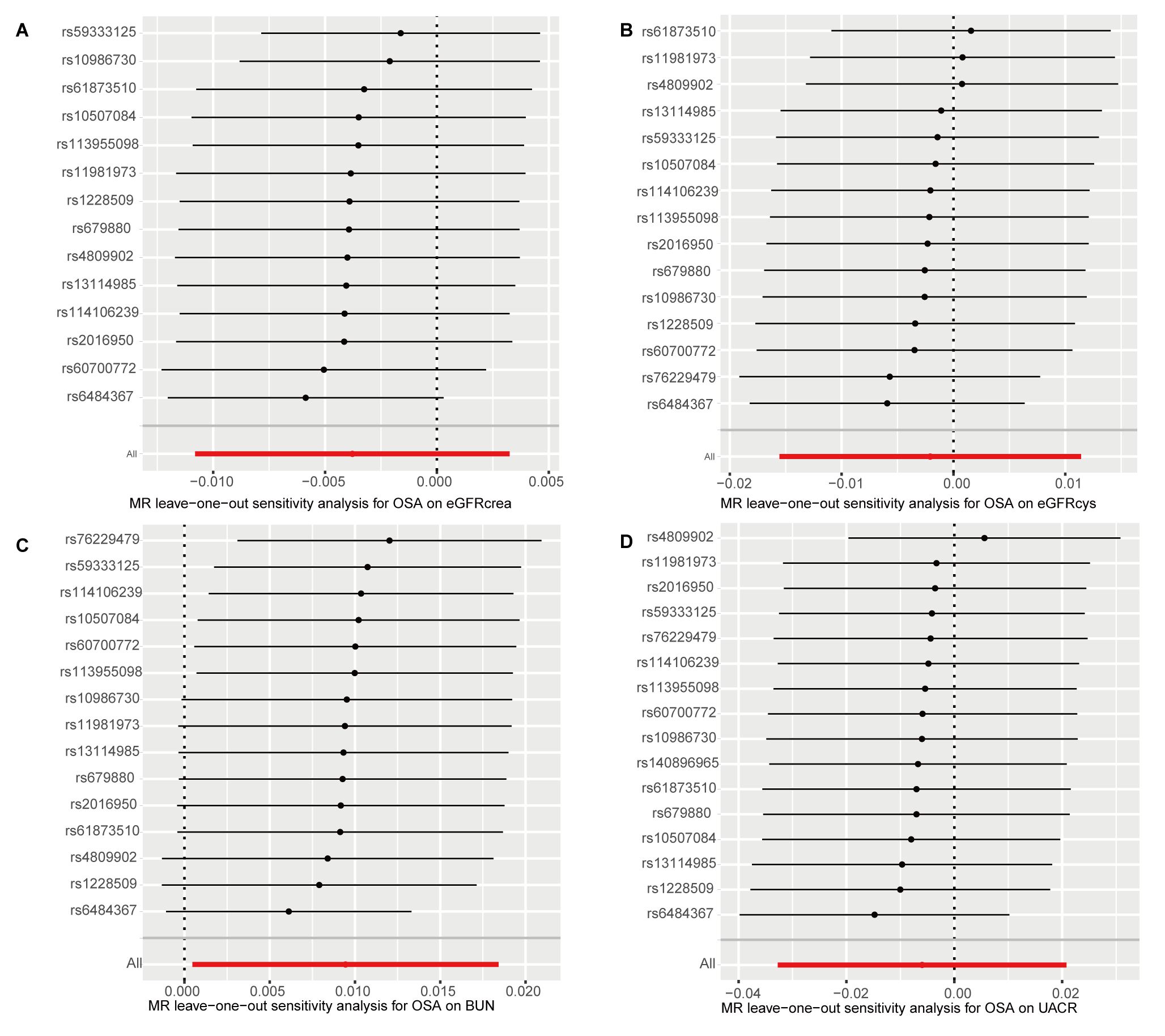
Supplement Figure 3. Leave-one-out analysis of the causal association between OSA and renal function phenotypes. (A)Leave-one-out analysis of the causal association between OSA and eGFRcrea; (B) Leave-one-out analysis of the causal association between OSA and eGFRcys; (C) Leave-one-out analysis of the causal association between OSA and BUN; (D) Leave-one-out analysis of the causal association between OSA and UACR.


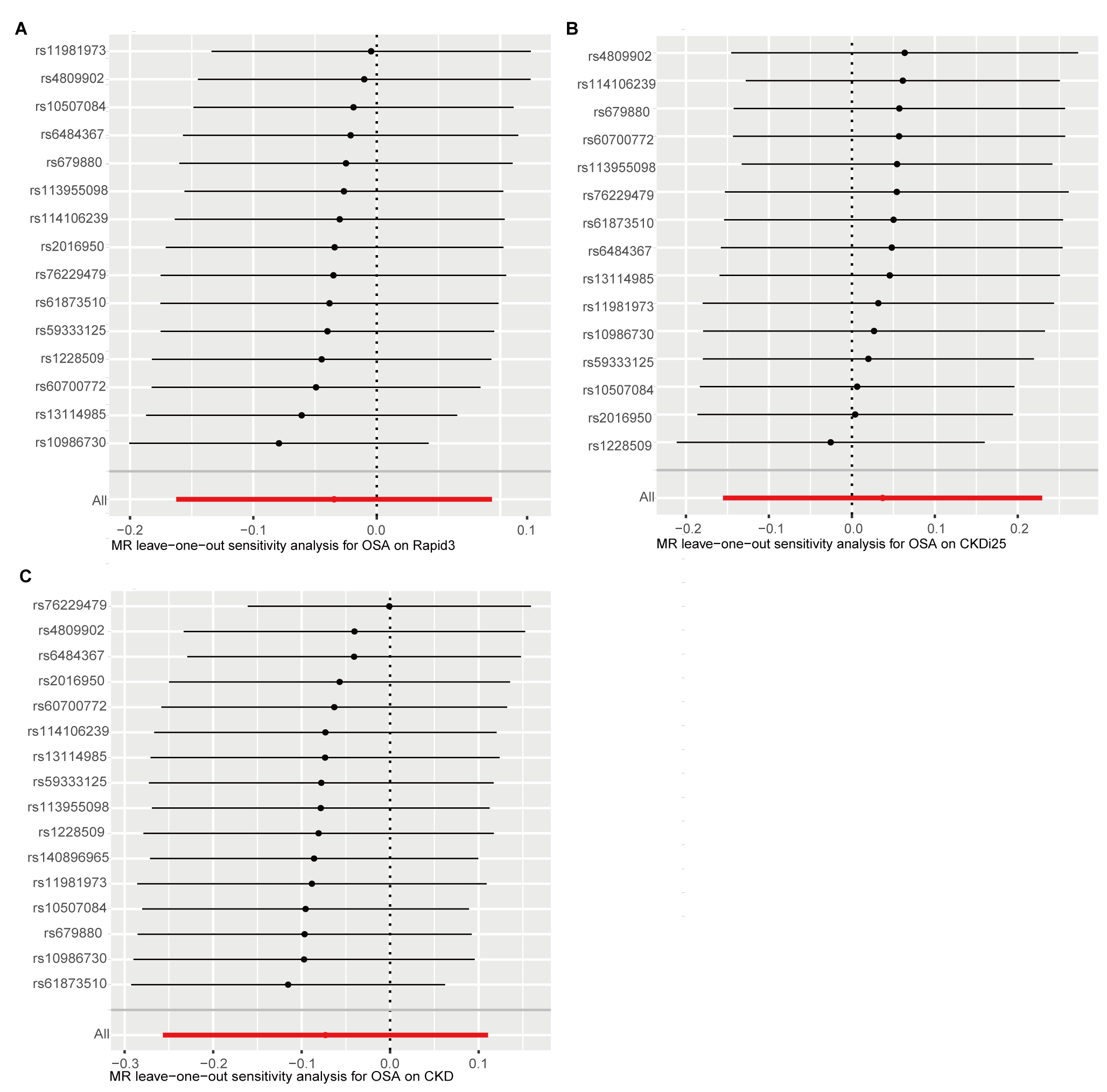
Supplement Figure 4.Leave-one-out analysis of the causal association between OSA and renal function phenotypes. (A) Leave-one-out analysis of the causal association between OSA and Rapid3; (B) Leave-one-out analysis of the causal association between OSA and CKDi25; (C) Leave-one-out analysis of the causal association between OSA and CKD.


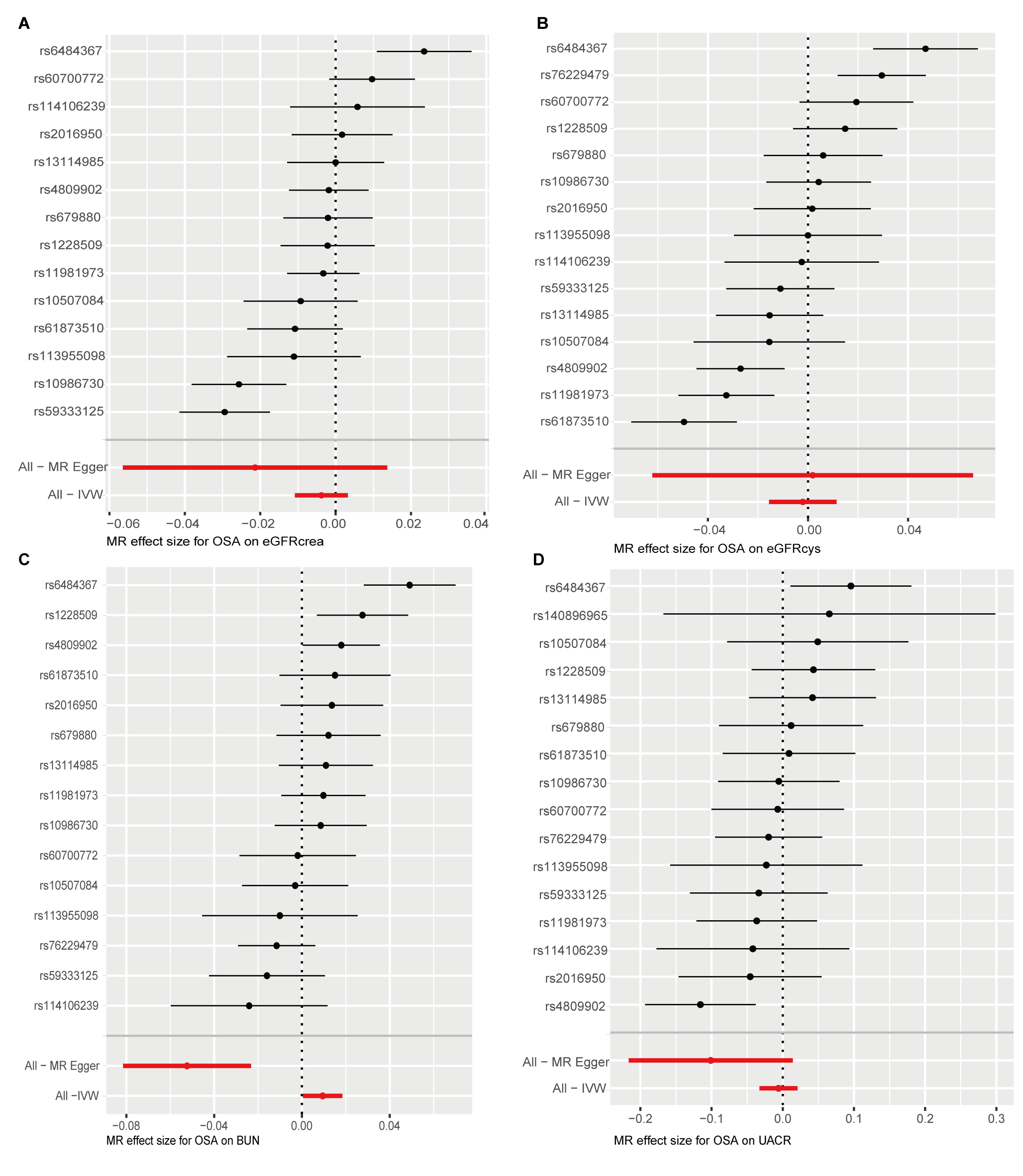


Supplement Figure 5. The forest plot of the causal effect of OSA on renal function phenotypes. The effect of each SNP was calculated separately, and the overall effect was calculated using MR Egger and IVW methods. (A)The forest plot of the causal effect of OSA on eGFRcrea; (B)The forest plot of the causal effect of OSA on eGFRcys; (C)The forest plot of the causal effect of OSA on BUN; (D) The forest plot of the causal effect of OSA on UACR.


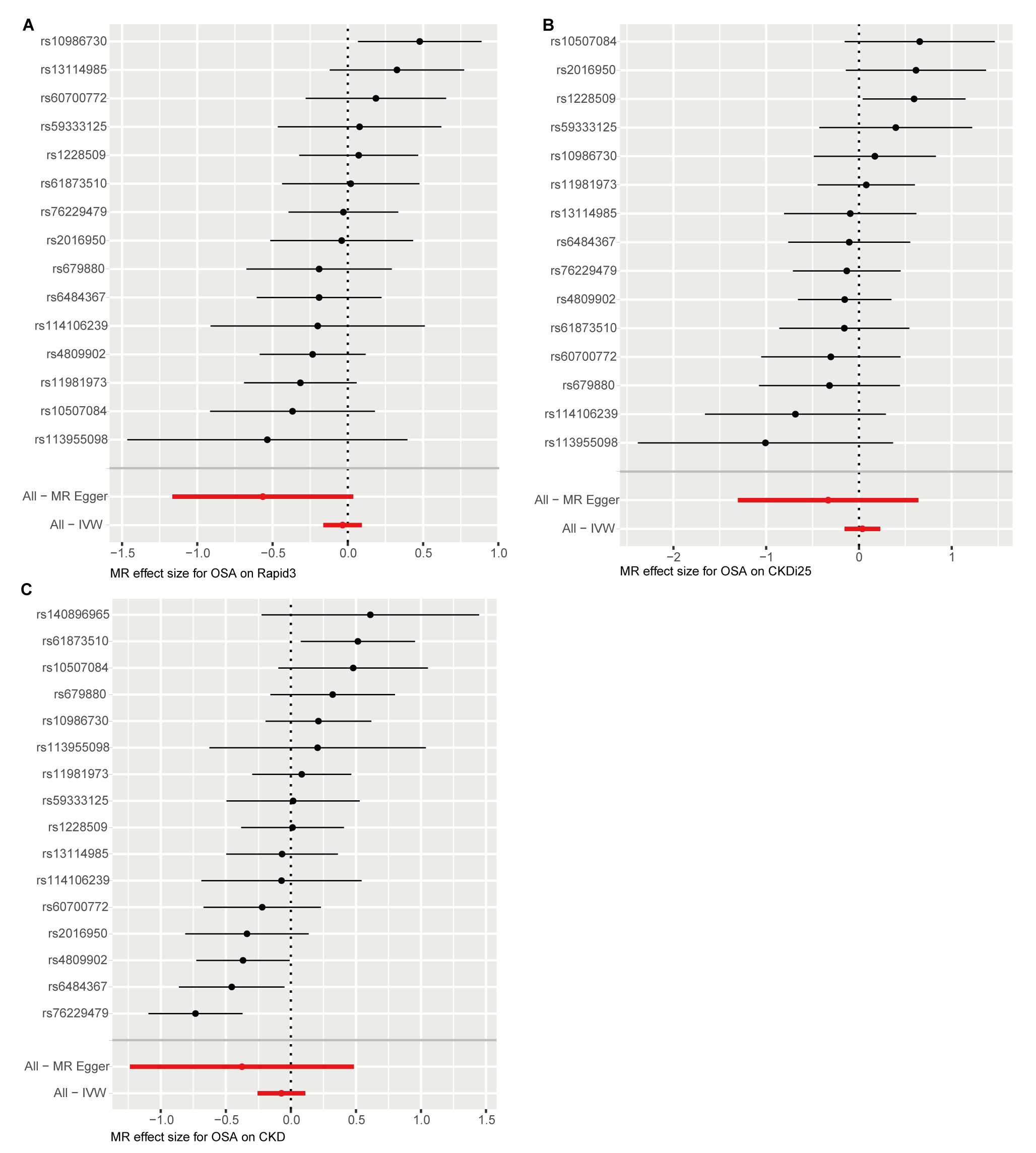


Supplement Figure 6. The forest plot of the causal effect of OSA on renal function phenotypes. The effect of each SNP was calculated separately, and the overall effect was calculated using MR Egger and IVW methods. (A)The forest plot of the causal effect of OSA on Rapid3; (B)The forest plot of the causal effect of OSA on CKDi25; (C)The forest plot of the causal effect of OSA on CKD.


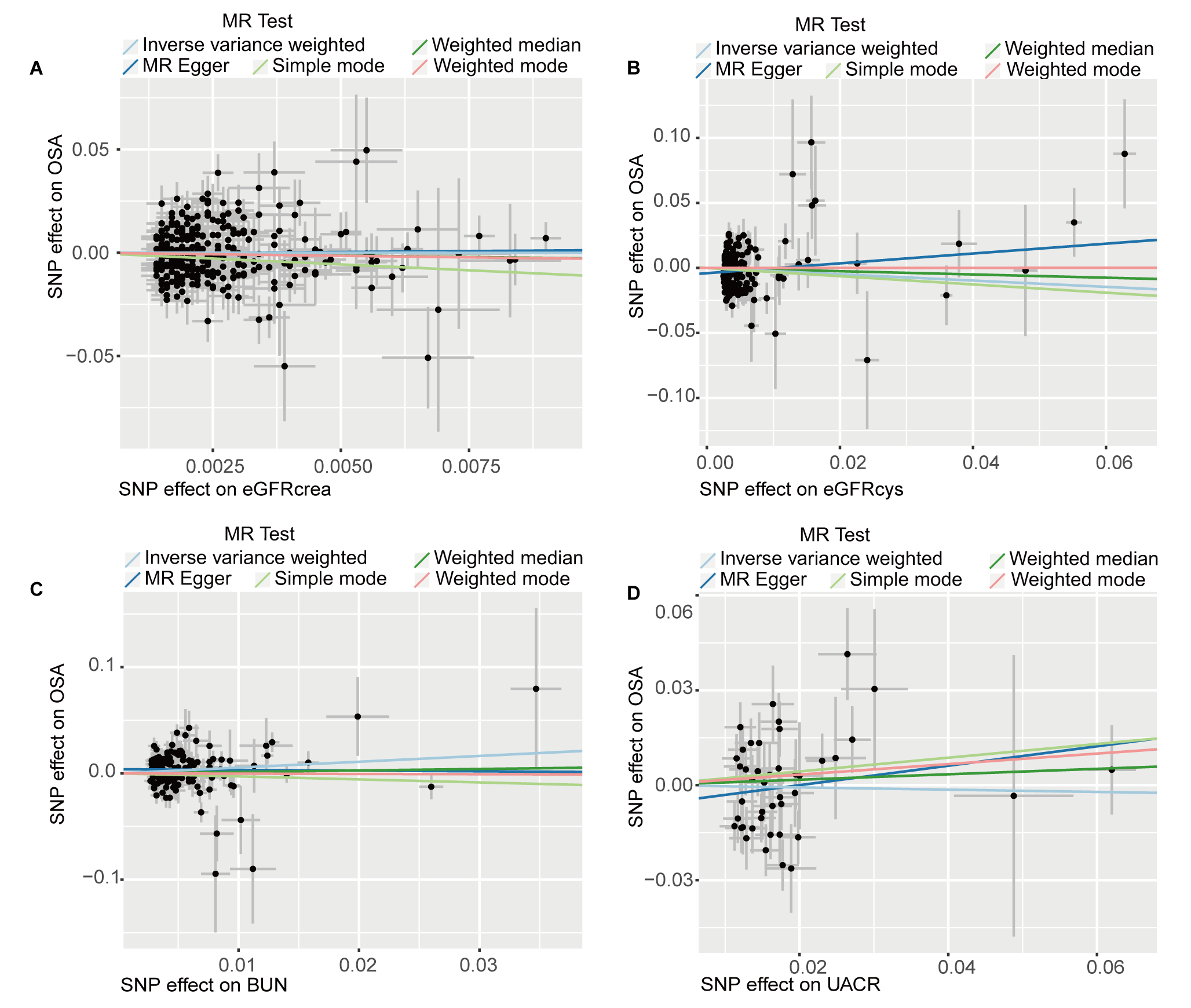


Supplement Figure 7. Scatter plot of the Mendelian randomization analysis results for the effect of the renal function phenotypes on OSA. (A) Causal effect of eGFRcrea on OSA using different MR methods; (B) Causal effect of eGFRcys on OSA using different MR methods; (C) Causal effect of BUN on OSA using different MR methods. (D) Causal effect of UACR on OSA using different MR methods.


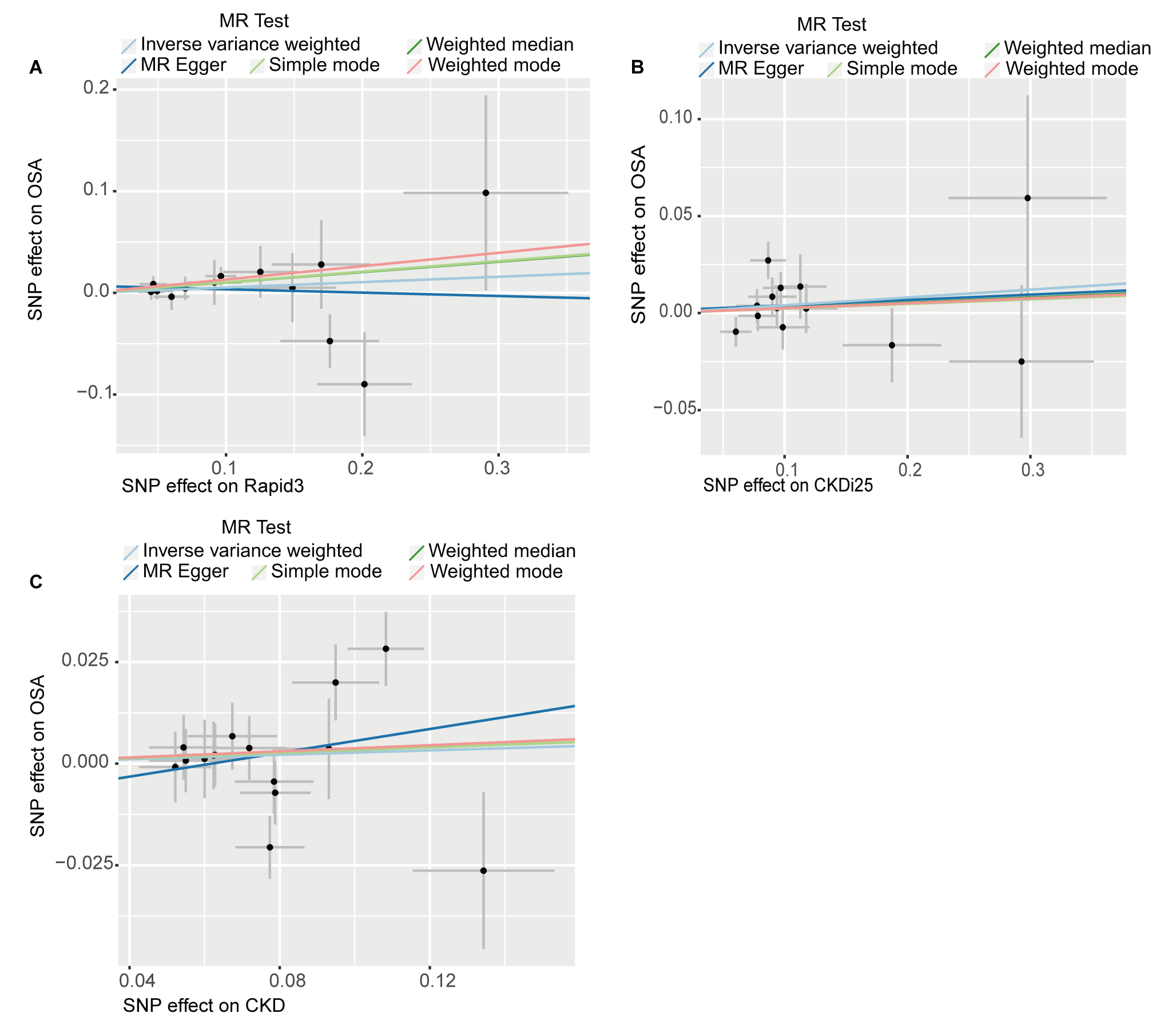


Supplement Figure 8. Scatter plot of the Mendelian randomization analysis results for the effect of the renal function phenotypes on OSA. (A) Causal effect of Rapid3 on OSA using different MR methods; (B) Causal effect of CKDi25 on OSA using different MR methods; (C) Causal effect of CKD on OSA using different MR methods.


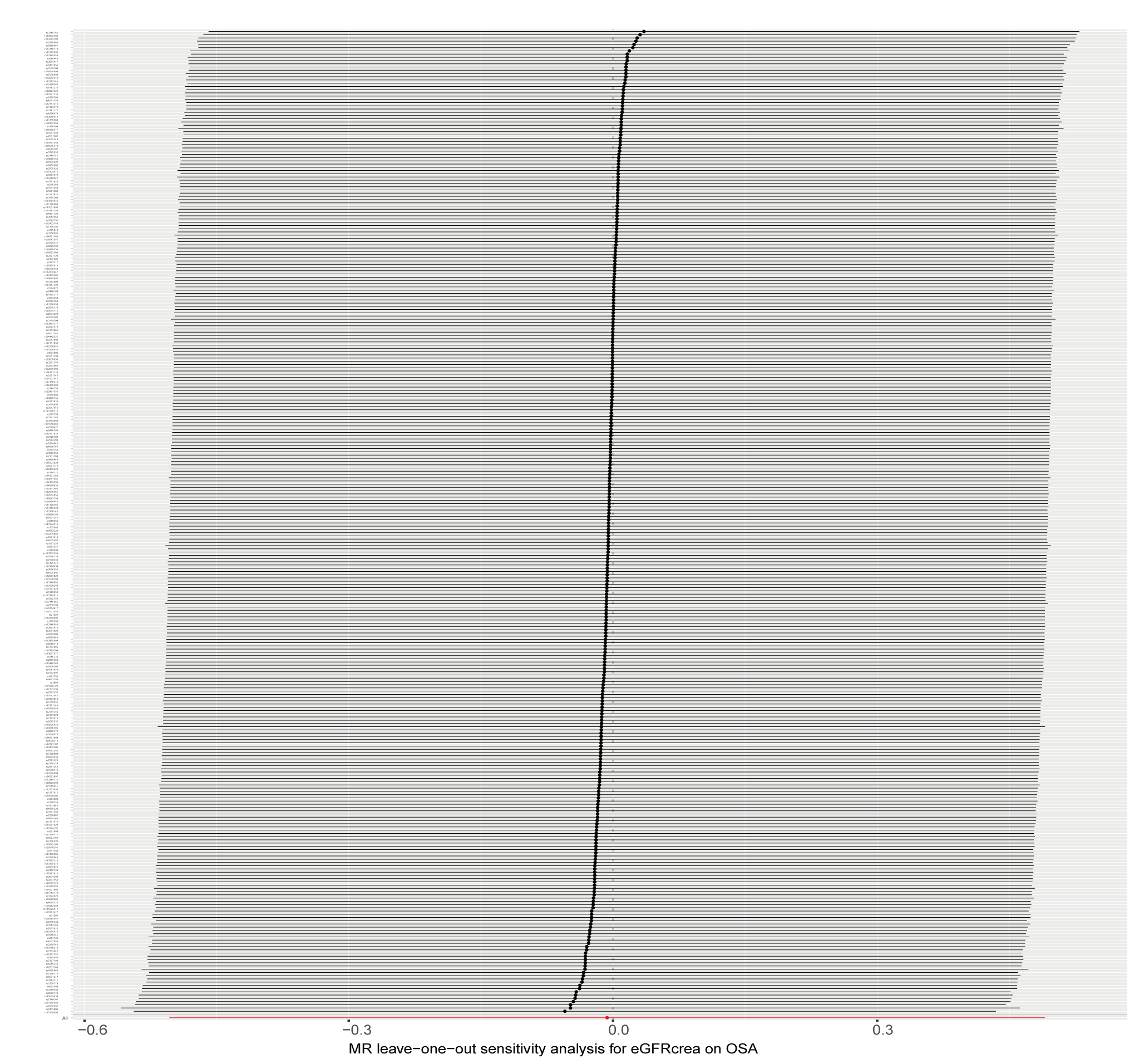


Supplement Figure 9. Leave-one-out analysis of the causal association between eGFRcrea and OSA.


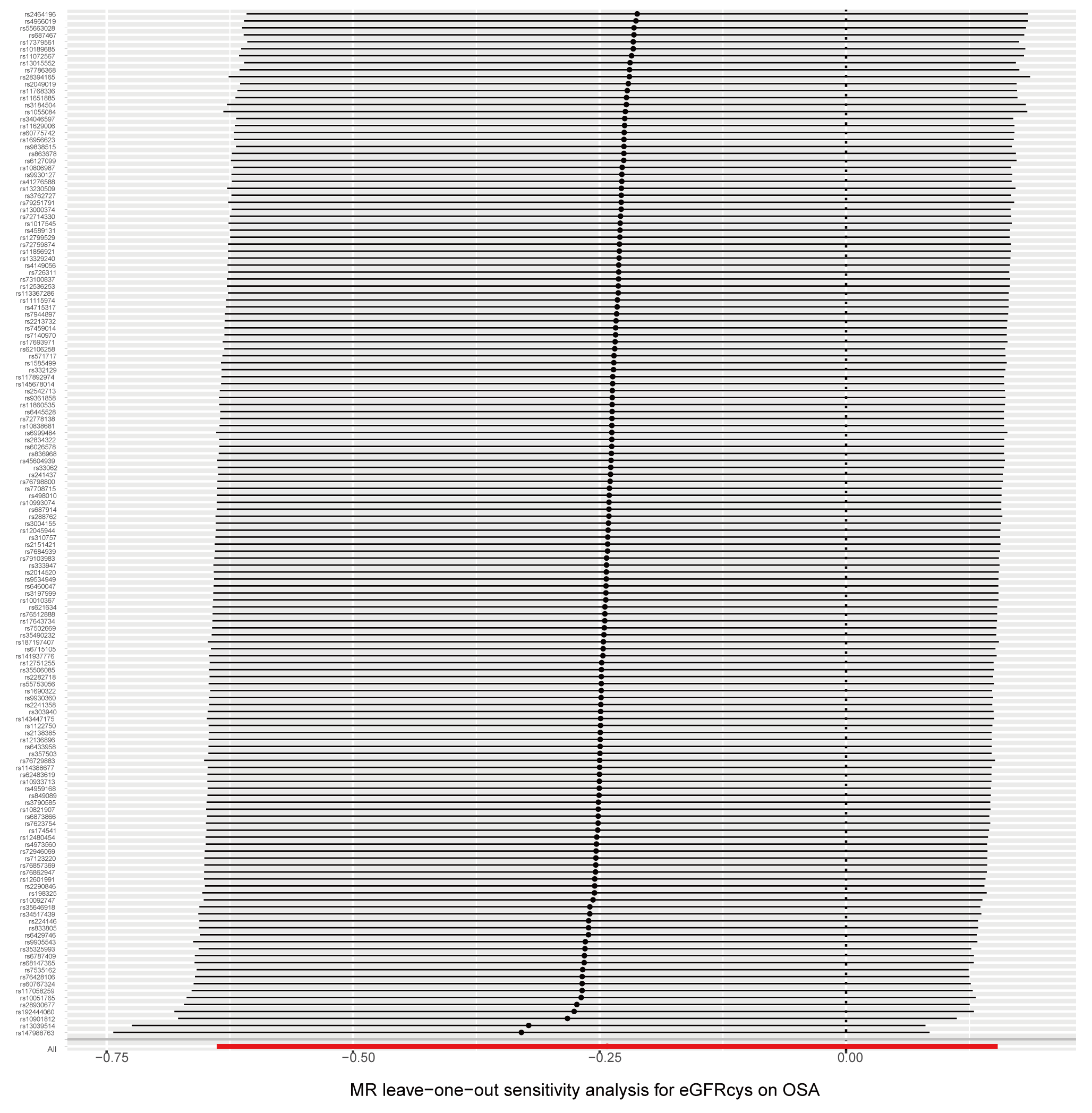


Supplement Figure 10. Leave-one-out analysis of the causal association between eGFRcys and OSA.


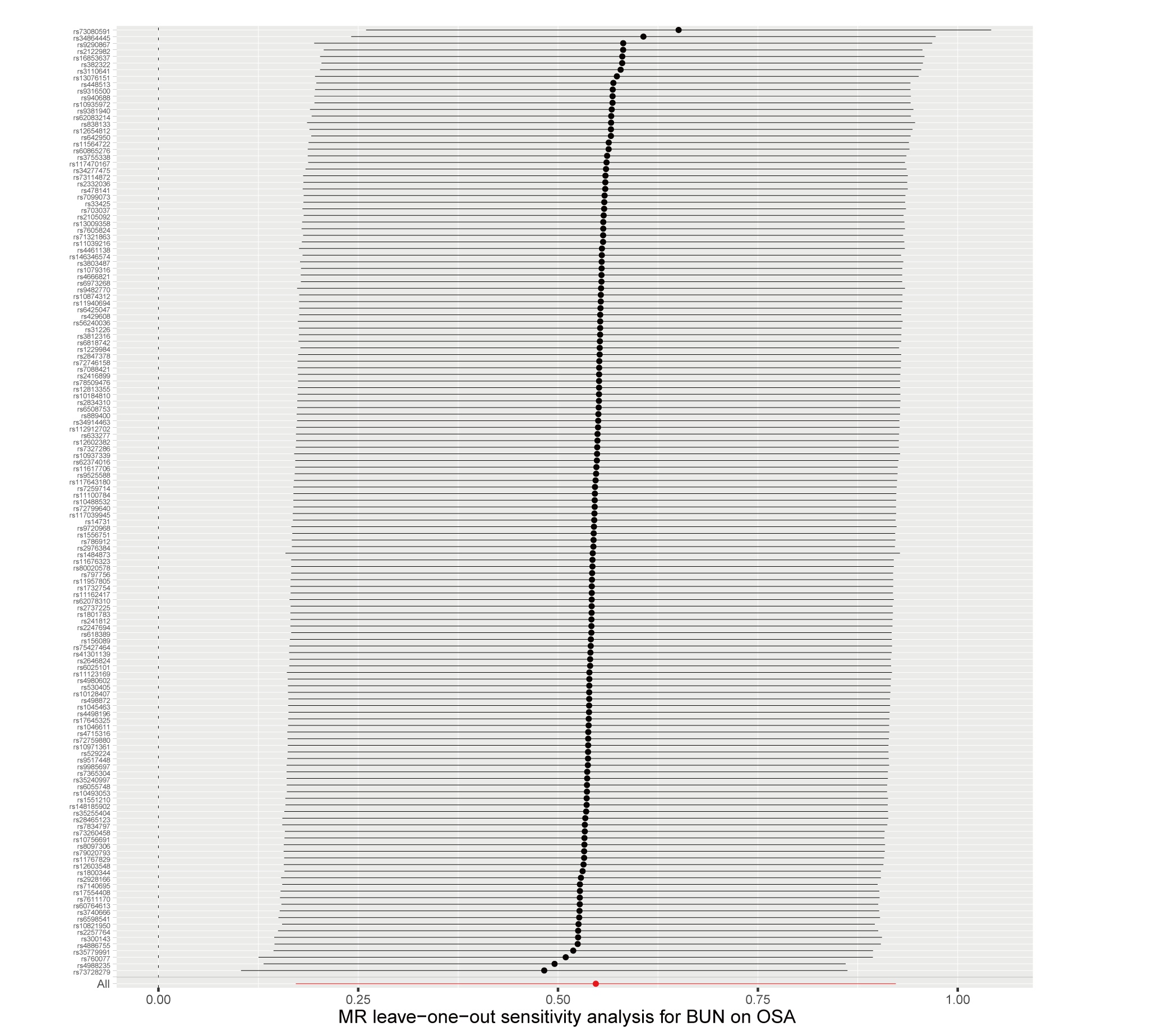


Supplement Figure 11. Leave-one-out analysis of the causal association between BUN and OSA.


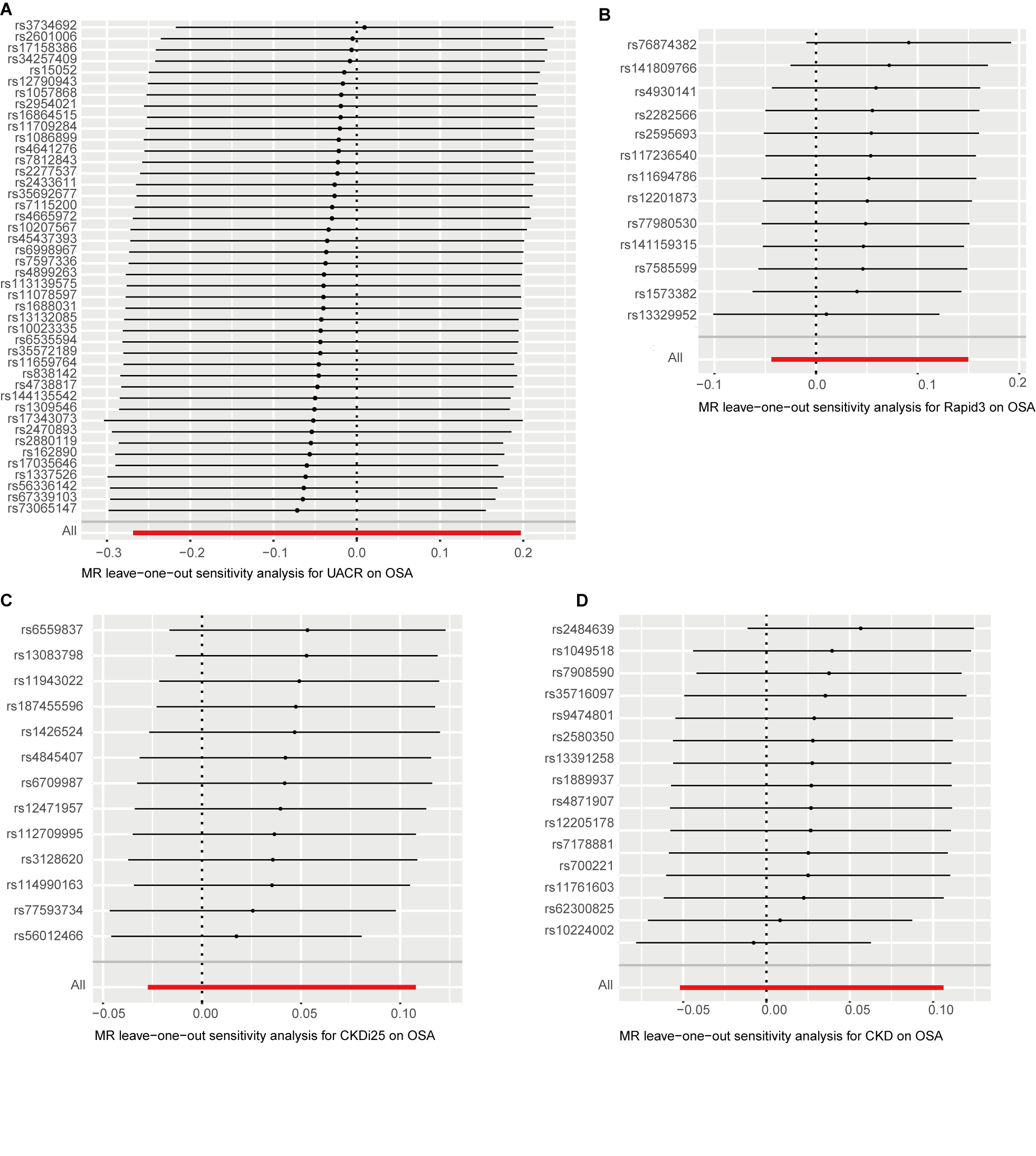


Supplement Figure 12. Leave-one-out analysis of the causal association between renal function phenotypes and OSA. (A) Leave-one-out analysis of the causal association between UACR and OSA; (B) Leave-one-out analysis of the causal association between Rapid3 and OSA; (C) Leave-one-out analysis of the causal association between CKDi25 and OSA; (D) Leave-one-out analysis of the causal association between CKD and OSA.


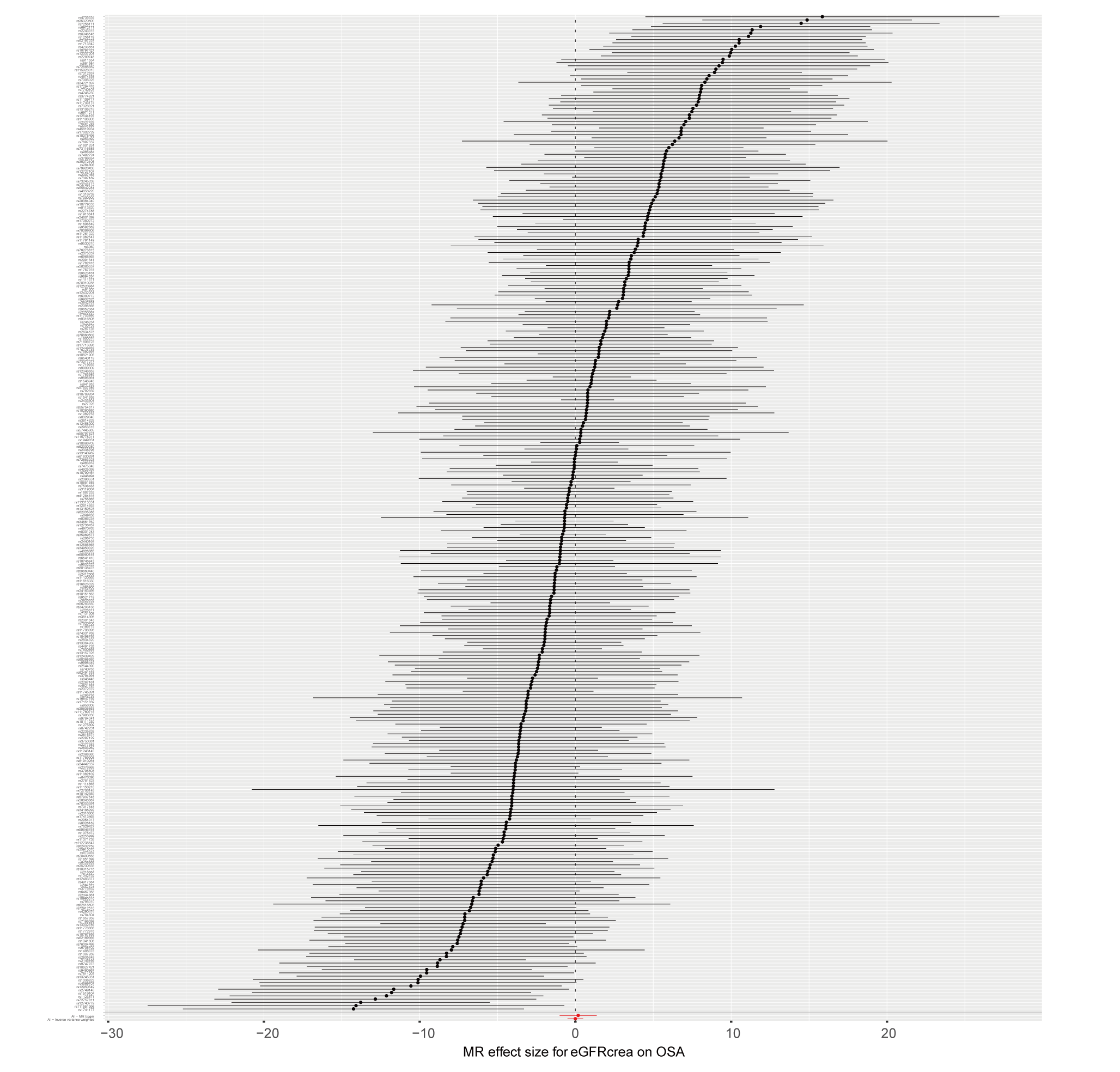


Supplement Figure 13. The forest plot of the causal effect of eGFRcrea on OSA. The effect of each SNP was calculated separately, and the overall effect was calculated using MR Egger and IVW methods.


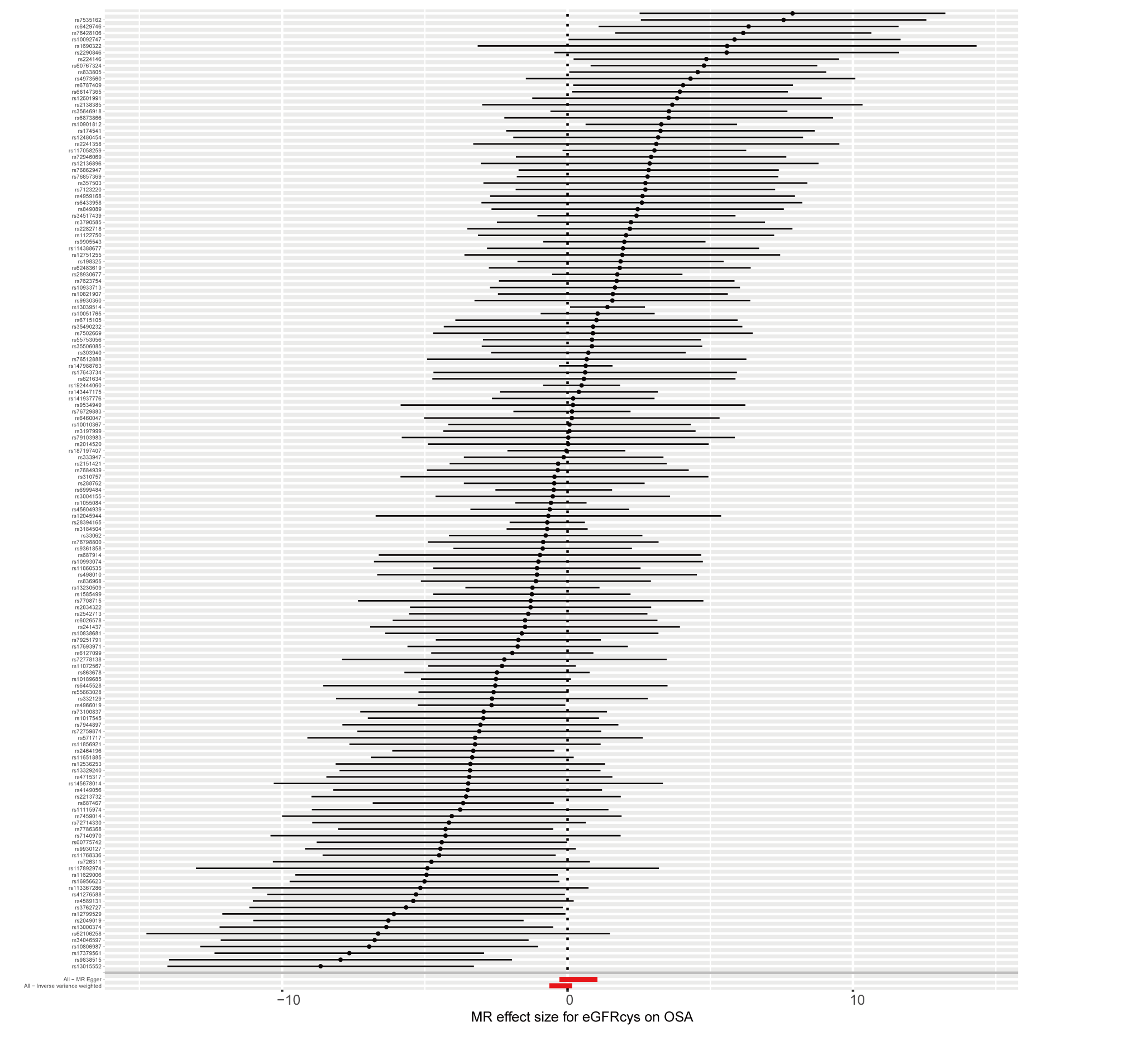


Supplement Figure 14. The forest plot of the causal effect of eGFRcys on OSA. The effect of each SNP was calculated separately, and the overall effect was calculated using MR Egger and IVW methods.


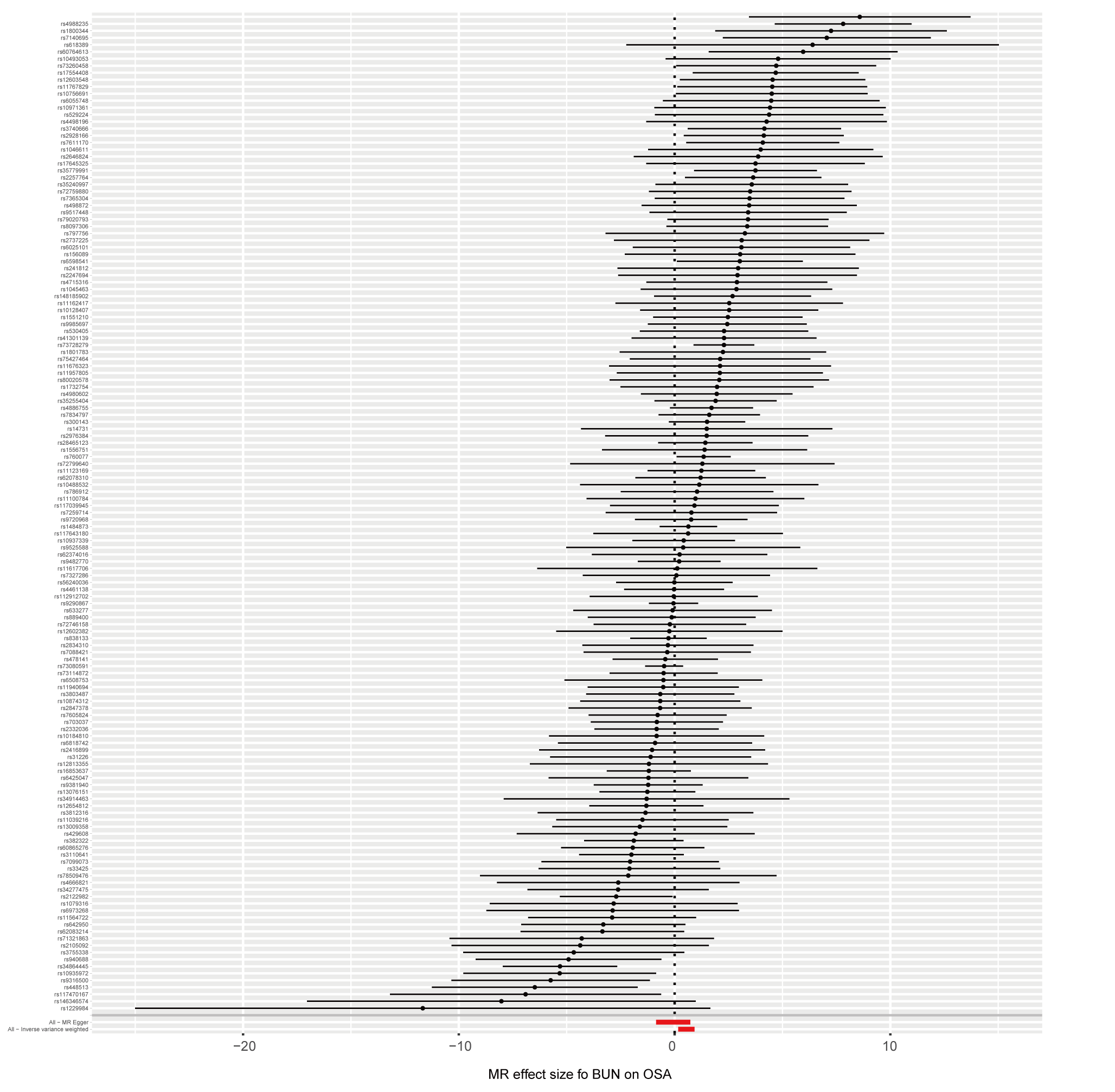


Supplement Figure 15. The forest plot of the causal effect of BUN on OSA. The effect of each SNP was calculated separately, and the overall effect was calculated using MR Egger and IVW methods.


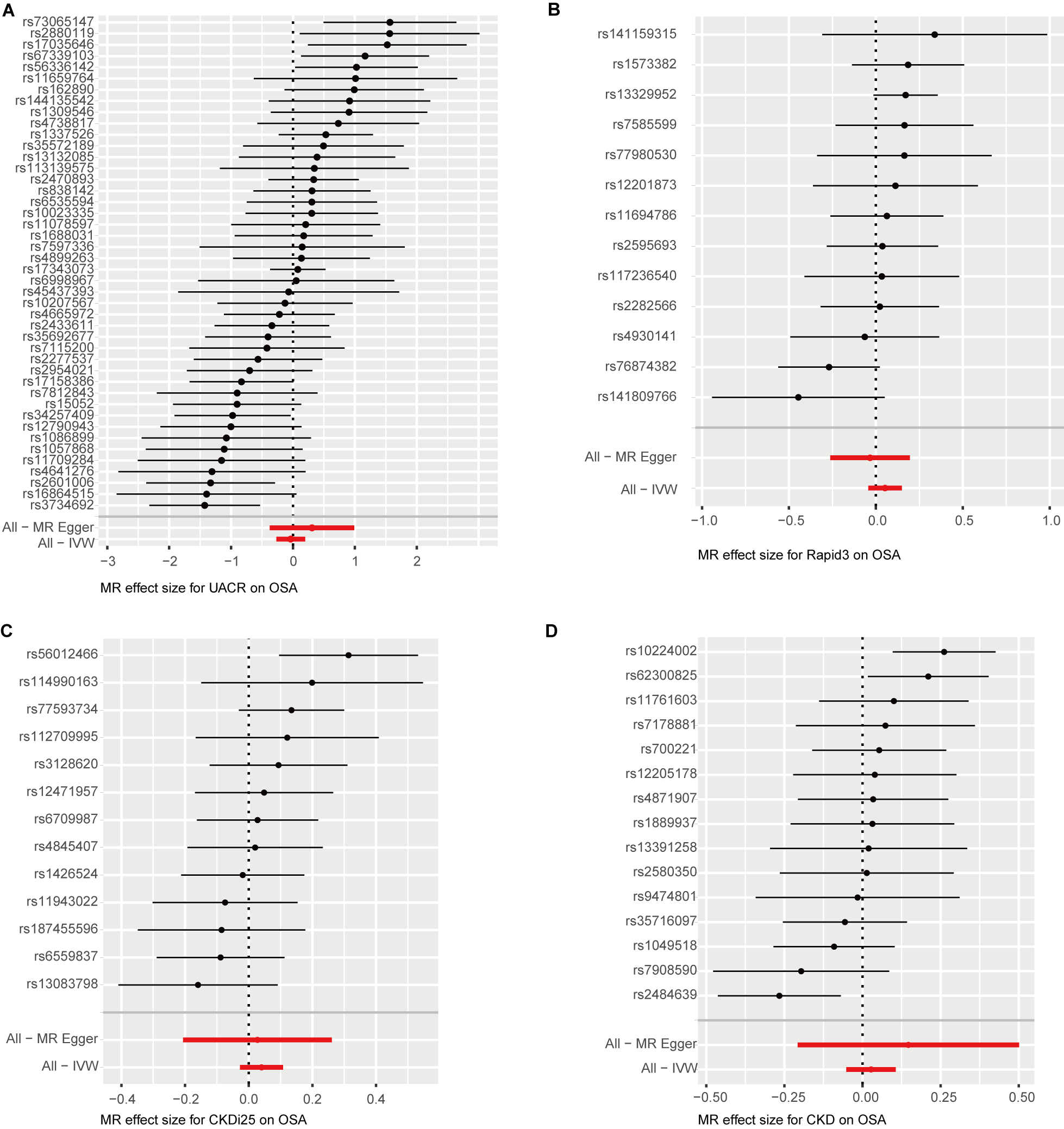


Supplement Figure 16. The forest plot of the causal effect of renal function phenotypes on OSA.The effect of each SNP was calculated separately, and the overall effect was calculated using MR Egger and IVW methods. (A) The forest plot of the causal effect of UACR on OSA; (B)The forest plot of the causal effect of Rapid3 on OSA; (C)The forest plot of the causal effect of CKDi25 on OSA; (D) The forest plot of the causal effect of CKD on OSA.
