# Supplementary material for: Causal effects of obstructive sleep apnea on chronic kidney disease and renal function: a bidirectional Mendelian randomization study
Source: Front Neurol. 2024 Sep 4;15:1323928. doi: 10.3389/fneur.2024.1323928 (PMC11408330; doi:10.3389/fneur.2024.1323928)
Supplement: Supplementary file 2 [file Table_2.DOCX]

Supplementary Table

Supplementary Table 1. Summary of genome-wide association studies (GWAS) datasets in our study.

| Phenotype | Abbreviation | Year | Consortium | Sample size | Reference genome | SNPs | Pubmed ID |
| --- | --- | --- | --- | --- | --- | --- | --- |
| creatinine-based estimated glomerular filtration rate | eGFRcrea | 2021 | CKDGen, UKB | 1,004,040 | GRCh37 | 8,844,847 | 34272381 |
| cystatinC-based estimated glomerular filtration rate | eGFRcys | 2021 | CKDGen, UKB | 852,678 | GRCh37 | 7,657,695 | 34272381 |
| blood urea nitrogen | BUN | 2021 | CKDGen, UKB | 460,826 | GRCh37 | 8,168,408 | 34272381 |
| urine albumin to creatinine ratio | UACR | 2019 | CKDGen | 547,361 | GRCh37 | 8,603,712 | 31511532 |
| rapid decline of eGFR | Rapid3 | 2021 | CKDGen, UKB | 141,964 | GRCh37 | 8,034,162 | 33137338 |
| rapid progress to CKD | CKDi25 | 2021 | CKDGen, UKB | 195,145 | GRCh37 | 8,641,125 | 33137338 |
| Chronic kidney disease | CKD | 2019 | CKDGen | 480,698 | GRCh37 | 9,162,323 | 31152163 |

Supplementary Table 2. Heterogeneity test and pleiotropy test.

| Exposure/Outcome | MR-IVW | | | MR-Egger | | | MR-Egger intercept | | |
| --- | --- | --- | --- | --- | --- | --- | --- | --- | --- |
|  | Q | Q_df | Q_pval | Q | Q_df | Q_pval | Intercept | SE | *P* val |
| OSA/ eGFRcrea | 57.327 | 13 | 1.57E-07 | 52.895 | 12 | 4.30E-07 | 9.74E-04 | 9.72E-04 | 0.336 |
| OSA/ eGFRcys | 80.06 | 14 | 2.76E-11 | 79.964 | 13 | 1.12E-11 | -2.26E-04 | 1.81E-03 | 0.903 |
| OSA/BUN | 33.292 | 14 | 0.003 | 13.955 | 13 | 0.377 | 3.48E-03 | 8.20E-04 | 9.58E-04 |
| OSA/UACR | 18.678 | 15 | 0.229 | 15.605 | 14 | 0.338 | 5.38E-03 | 3.24E-03 | 0.119 |
| OSA/Rapid3 | 16.931 | 14 | 0.260 | 13.659 | 13 | 0.398 | 0.029 | 0.017 | 0.101 |
| OSA/CKDi25 | 16.728 | 14 | 0.271 | 16.018 | 13 | 0.248 | 0.021 | 0.027 | 0.461 |
| OSA/CKD | 39.245 | 15 | 5.89E-04 | 37.892 | 14 | 5.40E-04 | 0.017 | 0.024 | 0.491 |
| eGFRcrea /OSA | 515.076 | 303 | 3.27E-13 | 514.893 | 302 | 2.59E-13 | -5.23E-04 | 1.60E-03 | 0.743 |
| eGFRcys/OSA | 288.771 | 146 | 1.94E-11 | 278.951 | 145 | 1.61E-10 | -4.04E-03 | 1.79E-03 | 0.025 |
| BUN/OSA | 282.244 | 142 | 2.49E-11 | 276.471 | 141 | 7.59E-11 | 3.89E-03 | 2.26E-03 | 0.088 |
| UACR /OSA | 92.704 | 43 | 1.66E-05 | 90.352 | 42 | 2.16E-05 | -6.15E-03 | 5.88E-03 | 0.302 |
| Rapid3/OSA | 12.387 | 12 | 0.415 | 11.68 | 11 | 0.388 | 6.88E-03 | 8.44E-03 | 0.432 |
| CKDi25/OSA | 14.882 | 12 | 0.248 | 14.865 | 11 | 0.189 | 1.28E-03 | 1.13E-02 | 0.912 |
| CKD/OSA | 24.891 | 14 | 0.036 | 24.039 | 13 | 0.031 | -9.07E-03 | 1.34E-02 | 0.509 |

Supplementary Table 3. Heterogeneity test and pleiotropy test after removing the outlier SNPs identified by Radial MR.

| Exposure/Outcome | MR-IVW | | | MR-Egger | | | MR-Egger intercept | | |
| --- | --- | --- | --- | --- | --- | --- | --- | --- | --- |
|  | Q | Q_df | Q_pval | Q | Q_df | Q_pval | Intercept | SE | *P* val |
| OSA/ eGFRcrea | 4.607 | 9 | 0.867 | 4.575 | 8 | 0.802 | -1.01E-04 | 5.60E-04 | 0.862 |
| OSA/ eGFRcys | 9.075 | 9 | 0.43 | 8.099 | 8 | 0.424 | 8.61E-04 | 8.76E-04 | 0.355 |
| OSA/BUN | 13.94 | 12 | 0.305 | 6.001 | 11 | 0.873 | 2.60E-03 | 9.24E-04 | 0.017 |
| OSA/CKD | 19.332 | 13 | 0.113 | 18.243 | 12 | 0.109 | -1.73E-02 | 2.05E-02 | 0.414 |
| eGFRcrea /OSA | 252.666 | 264 | 0.681 | 251.776 | 263 | 0.68 | -1.20E-03 | 1.27E-03 | 0.346 |
| eGFRcys/OSA | 138.923 | 120 | 0.114 | 136.31 | 119 | 0.133 | -2.29E-03 | 1.52E-03 | 0.134 |
| BUN/OSA | 128.98 | 120 | 0.271 | 125.678 | 119 | 0.32 | 3.54E-03 | 2.00E-03 | 0.080 |
| UACR /OSA | 44.903 | 35 | 0.122 | 42.796 | 34 | 0.143 | -5.98E-03 | 4.62E-03 | 0.204 |
| CKD/OSA | 8.562 | 12 | 0.74 | 8.428 | 11 | 0.675 | 4.15E-03 | 1.14E-02 | 0.722 |
